# Defining ethical standards for the application of digital tools to population health research

**DOI:** 10.2471/BLT.19.237370

**Published:** 2019-01-17

**Authors:** Gabrielle Samuel, Gemma Derrick

**Affiliations:** a Department of Global Health and Social Medicine, King's College London, Bush House, North East Wing, The Strand, London, WC2R 2LS, England.; b Department of Educational Research, Lancaster University, Lancaster, England.

## Abstract

There is growing interest in population health research, which uses methods based on artificial intelligence. Such research draws on a range of clinical and non-clinical data to make predictions about health risks, such as identifying epidemics and monitoring disease spread. Much of this research uses data from social media in the public domain or anonymous secondary health data and is therefore exempt from ethics committee scrutiny. While the ethical use and regulation of digital-based research has been discussed, little attention has been given to the ethics governance of such research in higher education institutions in the field of population health. Such governance is essential to how scholars make ethical decisions and provides assurance to the public that researchers are acting ethically. We propose a process of ethics governance for population health research in higher education institutions. The approach takes the form of review after the research has been completed, with particular focus on the role artificial intelligence algorithms play in augmenting decision-making. The first layer of review could be national, open-science repositories for open-source algorithms and affiliated data or information which are developed during research. The second layer would be a sector-specific validation of the research processes and algorithms by a committee of academics and stakeholders with a wide range of expertise across disciplines. The committee could be created as an off-shoot of an already functioning national oversight body or health technology assessment organization. We use case studies of good practice to explore how this process might operate.

## Résumé

La recherche sur la santé de la population à l'aide de méthodes fondées sur l'intelligence artificielle suscite un intérêt croissant. Ce type de recherche s'appuie sur une série de données cliniques et non cliniques pour prédire les risques sanitaires, par exemple en détectant les épidémies et en surveillant la propagation des maladies. Une part importante de cette recherche emploie des données issues des réseaux sociaux, appartenant au domaine public, ou des données secondaires anonymes relatives à la santé. Par conséquent, elles ne sont soumises à aucun contrôle de la part d'un comité d'éthique. L'usage et les règles déontologiques ont certes fait l'objet de discussions, mais l'attention portée à la gouvernance de l'éthique dans le cadre des recherches que des établissements d'enseignement supérieur ont menées sur la santé des populations reste minime. Pourtant, une telle gouvernance est essentielle pour que les spécialistes puissent prendre des décisions éthiques et garantir au public que les chercheurs agissent dans le respect de la déontologie. Nous proposons un processus de gouvernance éthique pour la recherche sur la santé de la population dans les établissements d'enseignement supérieur. Notre approche consiste à établir un rapport à la fin de la recherche, qui se concentre sur le rôle joué par les algorithmes d'intelligence artificielle dans l'accroissement de la prise de décisions. Le premier niveau de rapport pourrait comporter des registres nationaux accessibles selon le principe de science ouverte pour les algorithmes open-source ainsi que les données ou informations connexes développés durant la recherche. Le second niveau serait composé d'une validation sectorielle des algorithmes et processus de recherche par un comité d'universitaires et d'intervenants possédant une large gamme de compétences dans diverses disciplines. Ce comité pourrait être créé en tant que ramification d'un organisme national de surveillance déjà à l'œuvre, ou d'un organisme d'évaluation des technologies de la santé. Nous utilisons des études de cas pour identifier les bonnes pratiques et découvrir comment ce processus pourrait être appliqué.

## Resumen

Existe un interés creciente en la investigación sanitaria poblacional, que utiliza métodos basados en la inteligencia artificial. Dicha investigación se basa en una serie de datos clínicos y no clínicos para hacer predicciones sobre los riesgos sanitarios, como la identificación de epidemias y el seguimiento de la propagación de enfermedades. Gran parte de esta investigación utiliza los datos de las redes sociales de dominio público o los datos sanitarios secundarios anónimos y, por lo tanto, está exenta del escrutinio del comité de ética. Si bien se ha debatido sobre el uso y la regulación éticos de la investigación basada en tecnología digital, se ha prestado poca atención a la gobernanza ética de dicha investigación en las instituciones de enseñanza superior relacionadas con la salud poblacional. Esa gobernanza es esencial sobre cómo los académicos toman decisiones éticas y ofrece garantías al público de que los investigadores actúan de manera ética. Se propone un proceso de gobernanza ética para la investigación sanitaria poblacional en las instituciones de educación superior. El enfoque adopta la forma de una revisión una vez que la investigación ha sido completada, con especial atención a la función que los algoritmos de inteligencia artificial desempeñan en el aumento de la toma de decisiones. La primera fase de revisión podría consistir en la creación de repositorios nacionales de ciencia abierta para los algoritmos de código abierto y los datos o información afiliados que se desarrollen durante la investigación. La segunda fase consistiría en la validación de los procesos y algoritmos de investigación en un sector específico por parte de un comité de académicos y partes interesadas con una amplia gama de conocimientos especializados en todas las disciplinas. El comité podría crearse como una rama de un organismo nacional de supervisión ya en funcionamiento o de una organización de evaluación sobre tecnologías de la salud. Se utilizan estudios de casos de buenas prácticas para explorar cómo podría funcionar este proceso.

## ملخص

هناك اهتمام متنام بأبحاث صحة السكان، والتي تستخدم أساليب تعتمد على الذكاء الاصطناعي. تعتمد مثل هذه الأبحاث على مجموعة من البيانات السريرية وغير السريرية لوضع تنبؤات حول المخاطر الصحية، مثل تحديد الأوبئة ومراقبة انتشار الأمراض. تستعين الكثير من هذه الأبحاث بالبيانات من وسائل التواصل الاجتماعي في المجال العام، أو بالبيانات الصحية الثانوية مجهولة الهوية، وهي بالتالي معفاة من فحص لجنة الأخلاقيات. بينما تتم مناقشة الاستخدام والتنظيم الأخلاقي للأبحاث الرقمية، تم توجيه القليل من الاهتمام للحوكمة الأخلاقية لمثل هذه الأبحاث في مؤسسات التعليم العالي في مجال صحة السكان. مثل هذه الحوكمة ضرورية لكيفية اتخاذ العلماء لقرارات أخلاقية، وهي تمنح تأكيدًا للعامة بأن الباحثين يتصرفون على نحو أخلاقي. نحن نقترح عملية للحوكمة الأخلاقية لأبحاث الصحية السكانية في مؤسسات التعليم العالي. يأخذ هذا الأسلوب شكلاً للمراجعة بعد اكتمال البحث، مع التركيز بشكل خاص على الدور الذي تلعبه خوارزميات الذكاء الاصطناعي في تحسين صنع القرار. يمكن أن تكون الطبقة الأولى من المراجعة مستودعات وطنية علمية مفتوحة للعلوم لخوارزميات مفتوحة المصدر، والبيانات أو المعلومات ذات الصلة التي يتم تطويرها أثناء البحث. وتكون الطبقة الثانية هي التحقق من عمليات وخوارزميات البحث على مستوى القطاع، بواسطة لجنة من الأكاديميين وأصحاب المصلحة من ذوي الخبرة الواسعة في مختلف التخصصات. يمكن إنشاء هذه اللجنة كفرع خارجي لهيئة وطنية فاعلة للرقابة، أو كمؤسسة تقييم للتقنية الصحية. نحن نستعين بدراسات الحالة للممارسات الجيدة من أجل استكشاف كيفية تفعيل هذه العملية.

## 摘要

人们对使用人工智能方法的人口健康研究越来越感兴趣。这类研究利用一系列临床和非临床数据来预测健康风险，例如识别流行病和监测疾病传播。这项研究大多使用公共领域的社交媒体数据或匿名的二手健康数据，因此不受伦理委员会的审查。虽然数字化研究的伦理运用和监管一直是人们关注的焦点，但在人口健康领域，高等院校内对此类研究的伦理治理却鲜少关注。这种治理对于学者如何做出伦理决策至关重要，并向公众保证研究人员的行为符合伦理道德。我们提出了应用于高等院校人口健康研究的伦理治理过程。该方法在研究完成后采取审查的形式，特别关注人工智能算法在增强决策中的作用。第一层审查是全国性开放科学库，用于在研究过程中开发的开源算法和关联数据或信息。第二层是由具有跨学科广泛专业知识的学者和利益相关者组成委员会，对研究过程和算法进行特定部门验证。该委员会可以作为已经开始运作的国家监督机构或健康技术评估组织的分支机构。我们通过最佳实践的案例研究来探索此过程将如何运作。

## Резюме

Интерес к исследованиям здоровья на уровне популяций, применяющим методы, основанные на использовании искусственного интеллекта, неуклонно растет. В таких исследованиях различные клинические и неклинические данные используются для прогнозирования рисков для здоровья, например выявления эпидемий и мониторинга распространения заболеваний. Большинство исследований опираются на данные из социальных сетей, находящиеся в открытом доступе, или на анонимные второстепенные показатели здоровья населения, поэтому такие данные не рассматриваются комитетами по вопросам этики. В то время как вопросы этичного использования и регулирования исследований, проводимых на основе цифровых технологий, постоянно обсуждаются, этичному руководству такими исследованиями в учреждениях высшего образования, занимающихся вопросами здоровья населения, уделяется слишком мало внимания. Однако такое руководство крайне важно для принятия этичных решений научными сотрудниками и обеспечивает общественности уверенность в том, что исследователи соблюдают нормы этики. Авторы предлагают ввести процесс этического руководства исследованиями в сфере здравоохранения населения в высших учебных заведениях. Подход предлагает проведение изучения и анализа данных по окончании исследования, уделяя особое внимание тому, какую роль играют алгоритмы искусственного интеллекта в процессе принятия решений. На первом уровне изучение может проводиться с использованием национальных открытых научных репозиториев, содержащих алгоритмы с открытым кодом и аффилированные данные или информацию, полученную в ходе исследования. На втором уровне может осуществляться специализированная валидация процессов и алгоритмов, использовавшихся в научных исследованиях, комитетом из научных сотрудников и партнеров, обладающих обширными знаниями и опытом работы в различных областях. Комитеты могут создаваться на базе уже существующих национальных надзорных органов или организаций, занимающихся оценкой технологий в здравоохранении. Авторы приводят анализ конкретных примеров рекомендуемых норм, чтобы изучить возможность использования таких процессов.

## Introduction

The co-founder of the independent thinktank DataEthics has observed that, “data ethics is fashionable.”^1^ This is true: international attention in both the private and public sector is becoming focused on the ethical implications of using digital data and artificial intelligence in both the health and non-health arena. Such scrutiny includes questions around data governance, data minimization (only collecting and processing data which are needed for specific purposes) and protecting the privacy of data subjects; considerations of consent and trust; concerns associated with data accountability, transparency and explainability; as well as issues related to fairness, justice and bias in data sets. It is concerning, however, that discussions are lacking about how to update ethics governance in higher education institutions to move towards a shared understanding of ethics best practice for research that uses digital data.^2^ In the public sector, ethics governance in higher education is central to the way scholars make decisions about ethical value, and frames the norms of what becomes considered ethically acceptable by the whole research community. Ethics governance also assures the public that publicly-funded researchers are acting ethically and that higher education institutions can be trusted. These assurances are even more important in the realm of public health research, where the risk to society, in terms of predictions which could lead to health over-surveillance or inequity, are particularly high.

Ethical behaviour in higher education research means ensuring adherence to shared understandings of appropriate researcher practices throughout the research process from inception to dissemination and beyond. We have argued previously that such behaviour is reinforced through an informal, research-governed process, which we termed an ethics ecosystem.^3^ An ethics ecosystem is an interconnected network of researchers, research institutions and external bodies (publishers, funding bodies, professional associations and their policies) who participate equally in the promotion, evaluation and enforcement of ethically responsible research behaviour. However, the development of a stable ethics ecosystem in higher education institutions has become increasingly challenging in the areas of research which use innovative methods such as artificial intelligence. This is because questions remain about how to manage, process and interpret data predictions in an ethically responsible manner. As such, there are no shared norms and understandings of how to conduct such research ethically; traditional tools for ensuring ethical research behaviour that focus solely on consent and privacy are losing relevance because they are insufficient to deal with the range of ethical issues raised by digital research. As researchers strive to reach new shared understandings of ethical practice, a culture of personal ethics has emerged whereby researchers monitor their own (often different) decisions about how best to act ethically, without being subject to accountability or audit by other parts of the ethics ecosytem.^3^

Normally, research ethics committees in higher education institutions are tasked with the role of ensuring ethics best practice for all research involving human participants. However, at least in the United Kingdom of Great Britain and Northern Ireland, a large proportion of health research, which uses artificial intelligence-based methods to analyse data is exempt from ethics committee scrutiny (G Samuel, Department of Global Health and Social Medicine, King’s College London, England, author’s unpublished observations, 2019). This practice is particularly true for research methods, which include the use of, for example, social media data in the public domain, geolocation data and anonymous secondary health data for which a licensing agreement has been signed. This lack of ethics scrutiny is compounded by inconsistent ethics guidelines for publishing such research in international peer-reviewed journals.^3^

There is growing interest in population health research, which uses artificial intelligence-based methods. Such research draws on a range of clinical and non-clinical data to make predictions about health risks, such as identifying epidemics and monitoring disease spread. Any public health tool that uses such data could potentially be developed without systematic ethics oversight by a higher education institution, and (potentially) little reflection on the public interest or concomitant risks attached to making health predictions from the tool. Even when there is oversight from a research ethics committee, we observe that committee members often lack the experience or confidence regarding particular issues associated with digital research (G Samuel, Department of Global Health and Social Medicine, King’s College London, England, author’s unpublished observations, 2019).^4^ To address this gap, we propose a model for ethical scrutiny of artificial intelligence-based research in public health.

## Approaches to ethics oversight

Commentators are only recently beginning to assert the need for more agreed-upon frameworks for ethics governance in higher education institutions.^5^ Awareness of the issues has been driven to some extent by a recent international, high-profile example of inappropriate ethical behaviour by a researcher in a United Kingdom higher education institution. The researcher designed software which was used by a consulting firm to access personal data from millions of social media users without their consent and which was used for political advertising purposes.^6^ Questions remain, however, regarding what a framework for ethics governance would look like in practice, and how, in some jurisdictions, such a system would guard against ethics dumping (exporting unethical research practices, for example, unethical data processing) to countries where research ethics committee oversight is lacking. 

An approach to ethics oversight, which we focus on here, could be an extra layer of governance in the ethics ecosystem after the research has been conducted, that is, an ex-post review. This approach would be particularly important when the research involves artificial intelligence-based algorithms, which have a role in augmented decision-making. This is because many of the ethical concerns voiced about these technologies relate to their impact on society rather than to questions around the research itself. As we discuss below, the system would be analogous to premarket approval in drug regulation. Just as drug regulation aims to protect public health by ensuring the safety and efficacy of drugs, so too could ex-post review mitigate any societal harm potentially arising from artificial intelligence-based algorithms designed to make predictions about health. The review process would safeguard public health by minimizing the risk of harm caused, for example, by placing an over-reliance on artificial intelligence for decision-making in cases where it could potentially make inaccurate or unfair predictions. Such instances of harm have been reported in other sectors, and in the health sector the failure of Google Flu Trends reminds us to be cautious about the claims made for artificial intelligence.^7^^,^^8^ In this case, an algorithm was developed by Google to predict influenza outbreaks from the analysis of people’s searches for information assumed to relate to influenza on Google’s online search engine. The algorithm missed the peak of the 2013 influenza season in the United States of America because the search terms used in the construction of the algorithm produced inaccurate prediction results.^8^

The subject of ex-post review for oversight of the use of artificial intelligence in research is being discussed in the literature.^9^^–^^11^ Commentators have called for an artificial intelligence ombudsperson to ensure the auditing of allegedly unfair or inequitable uses of artificial intelligence. One proposed method is the adoption of trust labels, described as labels, which certify the trustworthiness of the algorithm, to ensure people understand the merits of artificial intelligence-based methods.^10^ Others talk about a potential licensing system to ensure quality control, analogous to licensing in many other sectors, such as in production and manufacturing.^11^

There have been doubts that any ex-post governance system can overcome the many obstacles required to cover the multiple evolving fields of digital research more generally.^11^ Nevertheless, a sector-specific^12^ and discipline-specific approach, particularly in health research, which is already well accustomed to such oversight and regulation, seems feasible at a national level. As higher education institutions have an emerging role in promoting their social role, oversight of artificial intelligence-based research is in line with recent principles underlying the practice of responsible research and innovation.^13^ We therefore believe that researchers should not retreat from the responsibility of ensuring such a system is established as part of the ethics ecosystem of higher education institutions.

## Proposed review model

The ex-post review model we propose is not a finished product, but a starting point to drive discussion within national public health and broader research communities towards committing to such an approach or a similar one. The model is two-layered, with ex-post review working at the second layer, though the system could potentially work with only the second layer.

The first layer of review would require a systematic, open-science infrastructure for centralized national repositories for open-source algorithms and affiliated data or information which are developed during the research process and which are intended for wide use beyond the research studies, particularly for decision-making. These repositories could be health-specific or non-health-specific, though the latter would be more feasible because the distinction between health and non-health research can be unclear and is not well defined within higher education institutions. A culture of open science and sharing of innovation is already promoted nationally and internationally,^14^ and expanding its remit for algorithms and associated workflows and software is not a daunting task.

Any open-science repository needs to be managed, curated and driven by higher education institutions and funding bodies, with clear incentives for compliance. For example, to develop best practice, there should be a requirement for algorithms and affiliated data to be placed in repositories just as there is for research data in many jurisdictions. We assume that in some instances access to data will need to be restricted to certain stakeholders, for certain data sets and certain circumstances. While this first layer is not a requirement for the second ex-post review layer, we believe that there are advantages to making artificial intelligence algorithms and the affiliated data more accessible. An open-science repository creates a way for other researchers and stakeholders to test the algorithms with their own data, checking for spurious predictions and highlighting any concerns or issues, which may be present within the artificial-intelligence prediction models. This open culture will have the added effect of driving innovation, because models can then be corrected and built on to achieve better predictions for the future.

The second layer of the ex-post review process would be a sector-specific validation of the research processes and algorithms. A sector-specific approach has also been suggested elsewhere,^15^ and makes sense because of its feasibility and ability to accommodate the specific needs of the health sector. These benefits mean that the review process can overcome some of the doubts raised about an ex-post governance system more generally, which would be difficult to implement across different sectors. A sector-specific approach also makes sense because regulatory systems are typically sector-specific. Consider the use of DNA (deoxyribonucleic acid) testing, for example, which is regulated differently in the criminal justice system than within health care and even wider society. Ex-post review would work best if it included the products of research not only from higher education institutions, but also private sector organizations. We propose such inclusion because much of the research we refer to is being carried out in the private sector and artificial intelligence algorithms developed in academia often require private-sector investment to make them feasible for wider public use. 

## Suggestions for implementation

Analogous systems of ex-post review of innovative prediction algorithms already exist in other sectors. In the Netherlands, for example, ex-post review is a legal requirement for the use of forensic DNA phenotyping in the criminal justice system. At present, if law enforcement officers have DNA samples of unknown origin from a crime scene, they can have the DNA tested (under specific circumstances) in such a way that makes predictions about certain externally visible characteristics of the person from whom the DNA originated. Legislation permits law enforcement officers to use only those tests which have been validated and reviewed and, at present, the law covers phenotype testing of hair and eye colour. For any new phenotype testing model to be considered for use, the system requires researchers to publish their validated models openly as a series of papers (similar to layer 1 of our model above). These papers are then submitted to the parliament of the Netherlands for review and for checking whether the models have been validated appropriately for use within the criminal justice system. Parliament therefore functions as an ombudsperson to provide sector-specific ex-post review of the testing models to ensure the underlying science is rigorous and validated.

Within the sphere of public health, ex-post review could be conducted by a committee comprising academics and stakeholders (such as lay people, professionals or users of the technology) with a wide range of expertise across disciplines, including but not limited to health, medicine, artificial intelligence research, social science and ethics. The aim of the committee would be to mitigate the risks of potential harm, which could be caused by the technology as much as possible by: reviewing scientific questions relating to the origin and quality of the data, algorithms and artificial intelligence; confirming the validation steps which have been conducted to ensure the prediction models work; and requesting further validation to be carried out, if required. This risk assessment could progress faster when researchers have already performed their own assessment of the societal impact of their research. As suggested elsewhere,^16^ this approach could also consider the integrity of participants’ data used in the research, and broader questions of social justice and value as they apply to the particular national jurisdiction. The committee could simply review or validate the research, have a more regulatory role or oversee any trialling of a research tool; in this way it would act as the artificial intelligence ombudsperson as discussed above. The committee may also be required to have a dynamic regulatory role, especially for algorithms whose performance evolves as more data are added to their systems. However, questions around the parameters of how this regulatory role could be put into practice still need answering, one suggestion being provisional licensing. One example of such an approach in the making is the International Telecommunication Union and World Health Organization focus group on artificial intelligence and health,^17^ which is working towards collecting a repository of data to test artificial intelligence technology for health within a standardized benchmarking framework.

At a national level, the establishment of a special committee might be burdensome in terms of slowing down the research process because it requires an additional element in the ethics ecosystem. Nevertheless, we argue that it is important to have a system of ethics governance when the research has a potentially high impact on the health of society. The costs could be minimized by streamlining the process. To add capacity, for example, the committee could be created as an off-shoot of an already functioning national oversight body or health technology assessment organization. The relevant organization would depend on the committees’ exact function in each jurisdiction. In the United Kingdom, for example, suitable candidates include the National Institute for Health and Care Excellence, the Medicines and Healthcare products Regulatory Agency or the newly founded Centre for Data Ethics and Innovation. All of these organizations are in the process of producing guidelines about the responsible use of artificial intelligence in health care. In fact, the United States Food and Drug Administration and the European Medicines Agency, which already regulate drug use in their respective territories via ex-post review, are being called upon to fulfil such a role for overseeing medical devices that use artificial intelligence in health care.^18^ Adding an additional division to focus specifically on the ex-post review of artificial intelligence-based research in the domain of public health would therefore be logical. Existing regulatory agencies are likely to be the most suitable candidates for such a role, and the European and United Kingdom’s agencies have now started introducing measures to scrutinize medical software. The measures include clarifying guidelines for medical devices in the light of digital data and software, and providing recommendations for the standardization of artificial intelligence in the medical device health-care sector. The Food and Drug Administration has now approved the first use of artificial intelligence to diagnose eye disease.^19^ Unclear boundaries over what constitutes a medical device, however, may leave some population-health specific algorithms outside of the remit of these organizations. Moreover, algorithms are context- and population-specific and will need validation in each national jurisdiction.

Together, national infrastructures, which incorporate ex-post review for artificial intelligence-based algorithms designed for health applications can start to re-balance the ethics ecosystem. In this way nations would begin the process of developing a new-shared understanding of ethics best practice for artificial intelligence-based public health research. Within this best practice, artificial intelligence-associated research will be openly scrutinized before any application that affects wider society is disseminated and used, to minimize as much as possible the potential for these systems to cause harm. Whether artificial intelligence-based health research continues to generate new ethical concerns or becomes just one more method in a researcher’s toolbox we must take care to avoid any unintended consequences of big data studies.
